# Non-mucinous, enteric-type thymic adenocarcinoma: genetic analysis of a case

**DOI:** 10.1186/s44215-026-00240-x

**Published:** 2026-02-02

**Authors:** Eiji Narusawa, Yoichi Ohtaki, Genichiro Ishii, Seshiru Nakazawa, Natsuko Kawatani, Tomohiro Yazawa, Kazuki Numajiri, Yuka Yoshida, Keisuke Nimura, Ken Shirabe

**Affiliations:** 1 Department of General Surgical Science, Division of General Thoracic Surgery, Integrative Center of General Surgery, Gunma University Graduate School of Medicine, Gunma University Hospital, 3-39-22 Showa-Machi, Maebashi, Gunma 371-8511 Japan; 2 Department of Thoracic Surgery, National Cancer Center Hospital East, Kashiwa, Japan; 3 Department of Pathology and Clinical Laboratories, National Cancer Center Hospital East, Kashiwa, Japan; 4 Department of Pathology, Gunma Saiseikai Maebashi Hospital, Maebashi, Gunma Japan; 5 Gunma University Initiative for Advanced Research, Gunma University, Maebashi, Japan

**Keywords:** Thymic carcinoma, Adenocarcinoma, Enteric-type thymic adenocarcinoma, TP53

## Abstract

**Background:**

Thymic adenocarcinoma is a rare histological subtype of thymic carcinoma. Non-mucinous enteric-type thymic adenocarcinomas are extremely rare.

**Case presentation:**

A 54-year-old woman with an abnormality detected on chest radiography was admitted to our hospital. Chest computed tomography showed a 5.5-cm-diameter mass in the anterior mediastinum. Blood carcinoembryonic antigen (CEA) level was highly elevated at 127 ng/ml (normal < 5), while other tumor markers, including alpha-fetoprotein, β-human chorionic gonadotropin, and interleukin-2R levels, were normal. Radiological findings suggested that the tumor was a thymic epithelium (Masaoka stage III). Surgery is performed for diagnostic and therapeutic purposes. Intraoperative findings revealed extensive pericardial invasion requiring a median sternotomy. The left brachiocephalic vein, pericardium, and lungs were resected along with the tumor to achieve complete resection.

Histological findings revealed that the tumor was composed of tall, columnar adenocarcinoma forming irregular lumina with no mucin production. Immunohistochemistry showed that cytokeratin 20 was partially positive and caudal type homeobox 2 was positive in approximately 50% of the tumor cells. Based on the morphological and immunohistochemical findings, enteric-type thymic adenocarcinoma was diagnosed per the 5th edition of the World Health Organization classification. The tumor was subtyped according to the Masaoka (stage IVB) and TNM classification criteria (T3N1M0 stage IVA). Plasma CEA levels decreased to normal levels after surgery. Further genetic analysis of the tumor revealed a pathogenic *TP53* stop-gain mutation (p.Arg213*), leading to the loss of p53 protein function. Postoperative adjuvant radiation therapy (54 Gy in 27 fractions) was administered under the suspicion of incomplete microscopic resection. Reportedly, the patient is in complete remission four years post-surgery.

**Conclusions:**

We encountered a rare case of non-mucinous enteric-type thymic adenocarcinoma harboring a pathogenic *TP53* mutation. Further studies are required to enunciate the features of this subtype of thymic carcinoma.

**Supplementary Information:**

The online version contains supplementary material available at 10.1186/s44215-026-00240-x.

## Background

Thymic carcinoma is a rare disease that accounts for approximately 14% of thymic tumors. Amongst thymic carcinoma, adenocarcinoma accounts for only 1.6% [1]. The fifth edition of the WHO reclassified the histologic types of thymic carcinoma into low-grade papillary adenocarcinoma; thymic carcinoma with adenoid cystic carcinoma-like features; enteric-type adenocarcinoma; and adenocarcinoma, NOS. Enteric-type adenocarcinomas are rare. Here, we report a case of non-mucinous enteric-type thymic adenocarcinoma with genetic alterations.

## Case presentation

**Fig. 1:**
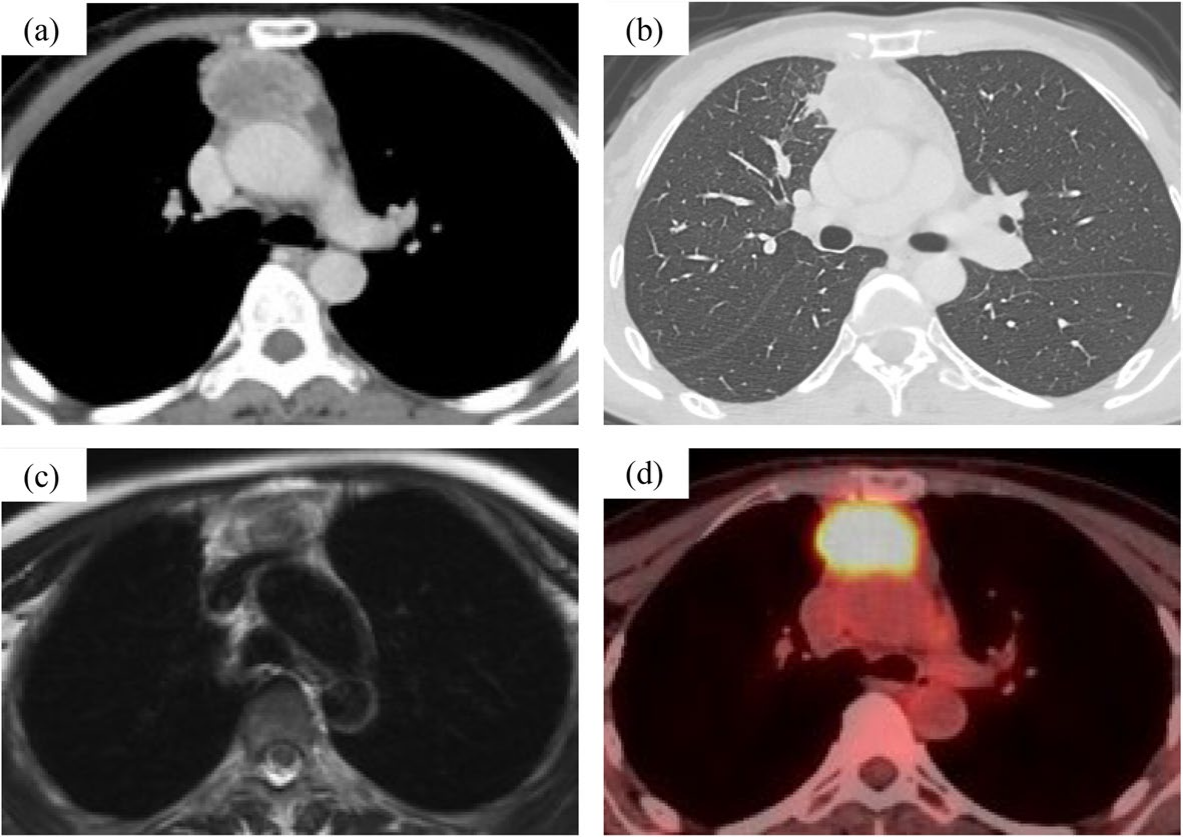
Radiological findings of the tumor (**a**) Chest computed tomography displays a 5.5-cm diameter mass in the anterior mediastinum. **b** The tumor directly invades the right upper lobe. **c** Magnetic resonance imaging reveals no obvious vascular invasion. **d** Fluorodeoxyglucose-positron emission tomography shows abnormal uptake in the mediastinal mass

A 54-year-old woman with an abnormality detected on chest imaging was admitted to our hospital. She had no history of smoking or malignancy. Chest CT revealed a 5.5-cm-diameter mass in the anterior mediastinum (Fig. 1a). The tumor was suspected to have directly invaded the right upper lung lobe (Fig. 1b). MRI showed no obvious invasion of the ascending aorta or left brachiocephalic vein (Fig. 1c). PET-CT showed fluorodeoxyglucose accumulation only in the mediastinal tumor, with a maximal standardized uptake value of 11.51, suggesting a primary thymic malignancy (Fig. 1d). Preoperative radiological findings revealed no metastases or malignancies of other origins. The blood CEA level was highly elevated at 127 ng/ml (normal, < 5 ng/mL), and the blood SCC, CYFRA, AFP, IL-2R, and β-hCG levels were normal. Based on the radiological and laboratory findings, we suspected a thymic epithelial tumor, especially thymic carcinoma (Masaoka stage III). The tumor was considered resectable and we decided to perform an initial surgery.

**Fig. 2:**
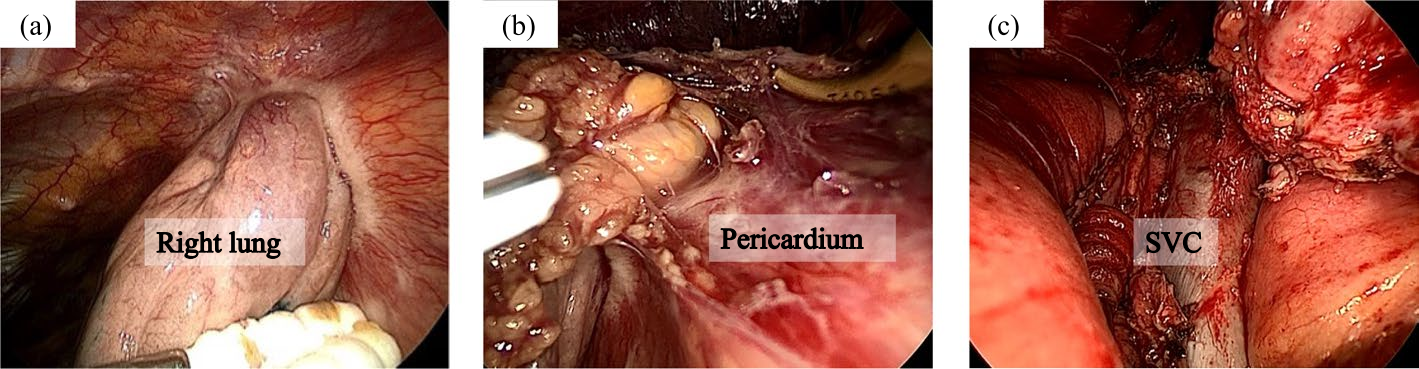
Intraoperative findings of the tumor. The tumor has extensively invaded the right lung (**a**) and pericardium (**b**). Median sternotomy is performed, which confirms that the tumor has invaded the left brachiocephalic vein and requires combined resection (**c**). SVC: superior vena cava

The patient was positioned supine with the right arm elevated. Initial assessment using a three-port thoracoscopic approach via the right hemithorax revealed invasion into the lung and pericardium. Therefore, we decided to resect the tumor using the median sternotomy approach (Fig. 2a, b). The pericardium and right lung were resected combined with the tumor, and the pericardium was reconstructed with a Gore-Tex® sheet. The left brachiocephalic vein was resected because of tumor invasion (Fig. 2c). Additionally, lymph node station 3a (prevascular, as defined by the IASLC lymph node map) was sampled for pathological evaluation. The surgical time was 228 min, and blood loss was 131 ml. The postoperative course was uneventful, and the patient was discharged on postoperative day 6.

**Fig. 3:**
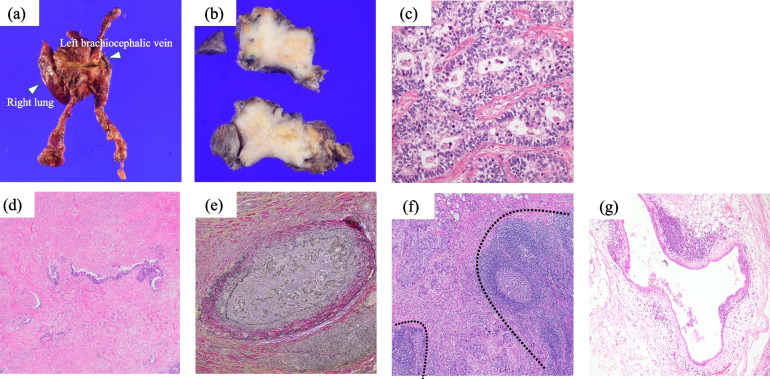
Pathological findings of the tumor. Macroscopically, the tumor extends directly to the right upper lobe, left brachiocephalic vein, and pericardium (**a**). The tumor is solid and cystic, and the cut surface is white and partly yellowish-white (**b**). Microscopic findings reveal a poorly differentiated carcinoma with a tubular structure. No papillary growth or differentiation into squamous cells is observed. **c** hematoxylin and eosin [HE], × 40), and severe fibrosis is observed around the tumor **d** HE staining, × 40) and vascular invasion **e** Elastica Van Gieson, × 40). The tumor directly invaded the adjacent lymph nodes **f** HE, × 40). Microscopic thymic cysts are found at the edge of the tumor **g** HE, × 10)

**Fig. 4:**
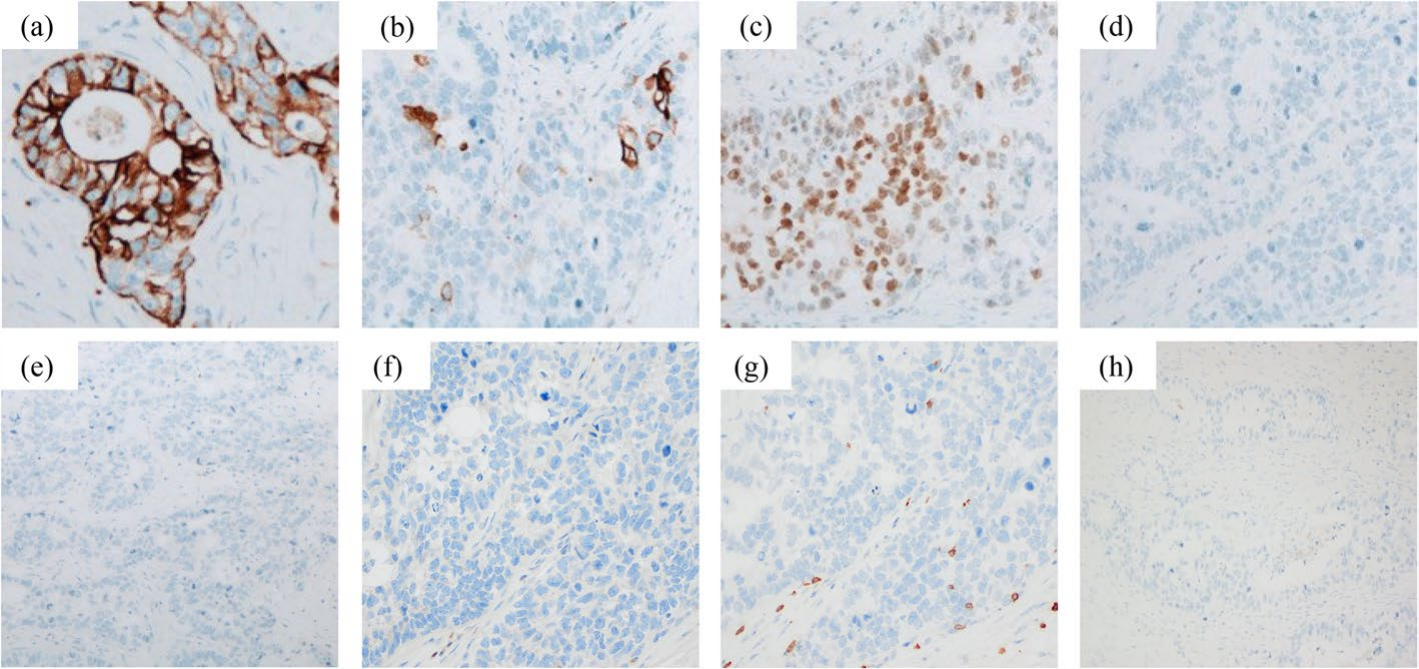
Immunohistochemical findings of the tumor. Immunohistochemical staining shows (**a**) positivity for cytokeratin (CK) 7, **b** partial positivity for CK20, **c** positivity for caudal type homeobox 2 (CDX-2), and negativity for (**d**) TTF-1, **e** p40, **f** kit, **g** CD5, and (**h**) MUC-2

Pathological findings are shown in Figs. 3 and 4. Macroscopically, the tumor measured 5.5 cm, and appeared as a solid mass with indistinct margins, containing central necrotic foci (Fig. 3a, b). Microscopically, tall columnar tumor cells with enlarged nuclei infiltrated and proliferated with glandular lumen formation (Fig. 3c). Severe fibrosis around the tumor (Fig. 3d) and vascular invasion (Fig. 3e). Pathological examination revealed tumor invasion of the brachiocephalic vein, pulmonary tissue, and pericardium (Supplementary Fig. 1). The tumor directly invaded the anterior mediastinal lymph nodes (Fig. 3f). Microscopic thymic cysts were observed at the edges of the tumor (Fig. 3g). Immunohistochemically, the tumor was positive for CK7, CK20, and CDX-2. Thyroid transcription factor 1, MUC-2, CD-5, and KIT were negative (Fig. 4). Differential diagnoses included the possibility of thymic metastasis from the gastrointestinal tract; however, preoperative radiological examinations revealed no diseases other than an anterior mediastinal tumor. The patient was diagnosed with enteric-type primary thymic adenocarcinoma (5th WHO Health Organization classification), Masaoka classification stage IVB, and TNM classification T3N1M0 stage IVA. Owing to the possibility of incomplete microscopic resection, postoperative adjuvant radiation therapy was administered (54 Gy in 27 fractions). The patient is alive without any recurrences four years postoperatively. The CEA level decreased to normal after surgery.

We performed a targeted gene mutation panel analysis (Axen Cancer Panel 1) on the resected tumor specimens (Supplementary Table 1). NGS analysis demonstrated a total read depth of 3,821 ×, mean read depth of 1,232 ×, and 99.8% of targeted bases covered at ≥ 100 ×. Non-synonymous SNVs and small indels meeting criteria of allele frequency ≥ 2% and read depth ≥ 100 × were selected for clinical interpretation. Of all 91 genes, a heterozygous *TP53* stop-gain mutation (c.637C > T; p.Arg213*) was identified as a pathogenic gene alteration at an AF of 19.7% (Table 1). Other detected missense variants were as follows; *ERBB3* (c.3380G > A, p.Arg1127His, AF 51.3%), *ERBB2* (c.428G > A, p.Arg143Gln, AF 41.3%), *FGFR1* (c.304G > A, p.Val102Ile, AF 43.4%), *BRCA2* (c.7378A > C, p.Asn2460His, AF 0.9%), and *MLH1* (c.1744C > G, p.Leu582Val, AF 51.2%). An in-frame insertion was detected in *AR* (c.234_239dupGCA, p.Gln79_Gln80dup, AF 100%). No actionable gene fusions or copy number alterations were detected (Table 1).

**Table 1:** Somatic variants detected in the tumor specimen by targeted next-generation sequencing

Gene	cDNA change	Protein change	Allele frequency (%)	Variant type	Clinical significance
TP53	c.637C > T	p.Arg213*	19.7	Stop gained	Pathogenic
ERBB3	c.3380G > A	p.Arg1127His	51.3	Missense variant	Likely benign
ERBB2	c.428G > A	p.Arg143Gln	41.3	Missense variant	Benign/Likely benign
FGFR1	c.304G > A	p.Val102Ile	43.4	Missense variant	Benign/Likely benign
AR	c.234_239dupGCA	p.Gln79_Gln80dup	100	Disruptive inframe insertion	Benign
BRCA2	c.7378A > C	p.Asn2460His	0.9	Missense variant	Uncertain significance
MLH1	c.1744C > G	p.Leu582Val	51.2	Missense variant	Conflicting interpretations of pathogenicity

Variants include pathogenic, variants of uncertain significance (VUS), and benign/likely benign mutations. This table details the genetic information, cDNA and protein alterations, allele frequency (percentage and alt/total reads), variant type, and clinical significance

## Discussion

Herein, we report an extremely rare case of primary thymic non-mucinous enteric-type adenocarcinoma harboring a TP53 mutation.

Enteric-type thymic adenocarcinoma is rare [2], and only 35 cases of thymic enteric-type adenocarcinoma have been reported to date (Supplementary Table 2). Moreover, there have been only 11 cases of non-mucinous enteric-type thymic adenocarcinoma, including our case [3–11]. The mean age of the patients was 51 years, and the cohort consisted of six men and five women. According to the Masaoka classification, two cases were stage I, three were stage II, two were stage III, and four were stage IVb. Elevated serum CEA levels were observed in three patients, while five had normal CEA levels. Surgical resection was performed in eight cases, with additional radiotherapy and/or chemotherapy administered as appropriate.

Generally, enteric-type adenocarcinomas exhibit colorectal features, such as high columnar cell morphology and intestinal-type glandular epithelial tumor cells growing in a papillary tubular fashion while forming tumor nests. Thymic adenocarcinomas are classified as enteric type if they are positive for at least one of the following immunohistochemical markers: CDX-2, MUC-2, or CK20. In addition, CK7 and CD5 may or may not be positive, whereas TTF-1 and c-KIT are negative [12]. In this case, adenocarcinoma forming irregular lumen was observed in some areas, however mucus was not observed, and CK20 was partially positive, and CDX-2 was positive in approximately 50% of the tumor cells. Because primary thymic adenocarcinoma is extremely rare, it is essential to exclude metastases from other organs [13, 14]. CK7 is often positive in thymic carcinoma, but is typically negative in colorectal carcinoma [15, 16]. In the present case, CK7 was positive, which helped to rule out a colorectal origin. Furthermore, there were no other findings suggestive of a primary tumor on CT or PET-CT, which led to the diagnosis of primary adenocarcinoma of the thymus.

As shown in Fig. 3g, more than half of patients with enteric-type adenocarcinomas had thymic cysts [5]. There are two hypotheses to explain the association between enteric-type thymic adenocarcinomas and thymic cysts. One is the malignant transformation of the preexisting cyst epithelium, and the other is a secondary cyst change associated with epithelial proliferation in response to tumor antigens [17, 18].

Preoperative MRI showed tumor contact with the left brachiocephalic vein, with a largely preserved fat plane and no definitive radiological evidence of invasion. However, pathology confirmed adventitial invasion, suggesting that routine MRI can overlook subtle venous involvement [19]. Furthermore, intense fluorodeoxyglucose uptake can also serve as a marker of invasion into adjacent structures; therefore, careful attention is warranted when high uptake is observed, as in the present case [20].

In the current case of primary thymic enteric-type adenocarcinoma, we identified a pathogenic *TP53* mutation, as well as other histological types of thymic carcinoma. In thymic carcinoma, including squamous cell carcinoma, TP53 mutations are among the frequently observed genetic alterations, with frequencies reported in approximately 7 to 25% of cases. Hotspot mutations in TP53 are thought to be key alterations in tumor development and progression [21].

Other common molecular abnormalities include inactivation of CDKN2A, which has been detected in as many as 40% of cases, and CDKN2B, found in up to 25%. Mutations in the KIT gene have also been reported, occurring in approximately 6 to 12% of cases [22]. Conversely, reports on genetic mutations in thymic adenocarcinomas are limited. To date, three cases of mucinous enteric-type thymic adenocarcinomas with genetic alterations have been reported. One patient harbored a KRAS mutation [23], another demonstrated an STK11/LKB1 mutation [24], and a third showed a TP53 mutation along with amplification of the MYC gene [25]. Notably, there are no reports on the genetic analysis of non-mucinous enteric-type thymic adenocarcinoma, and this is the first report of genetic alterations in non-mucinous enteric-type thymic adenocarcinoma. The TP53 mutation (p.Arg213*) identified in this study is believed to significantly impair protein function. Generally, truncating mutations in TP53 result in loss of tumor suppressor function and are regarded as pathogenic. Although data specific to thymic adenocarcinoma are limited, studies of thymic carcinoma have consistently shown that TP53 abnormalities are associated with higher malignancy, recurrence rates, and poorer survival outcomes [22].

In terms of oncologic outcome according to adenocarcinoma subtypes, Ahmad et al. reported no significant difference in recurrence-free or overall survival based on histological type [1], whereas Jung et al. demonstrated that enteric-type thymic carcinoma had a relatively better median survival (85.6 months) compared to non-enteric types (22.1 months) [7]. Kwon et al. further reported a poor prognosis in cases classified as NOS, although the number of cases was too small to establish definitive differences [4].

## Conclusions

We encountered a rare case of non-mucinous enteric-type thymic adenocarcinoma. Because reports on the genetic characteristics and oncological outcomes of enteric-type thymic adenocarcinoma are limited, further accumulation of genetic and clinical data is essential.

## Supplementary Information

Supplementary Material 1.

Supplementary Material 2.

## Data Availability

The dataset supporting the conclusion of this article is included within the article and its additional file.

## Abbreviations

WHO: World Health Organization
NOS: Not otherwise specified
CT: Computed tomography
MRI: Magnetic resonance imaging
PET: Positron emission tomography
β-hCG: β-Human chorionic gonadotropin
CK: Cytokeratin
CDX-2: Caudal type homeobox 2
IL: Interleukin
NGS: Next generation sequencing
SNVs: Single nucleotide variants
AF: Allele frequency
PORT: Postoperative adjuvant radiation therapy
SCC: Squamous cell carcinoma
CYFRA: Cytokeratin 19 fragment
AFP: Alpha-fetoprotein
IASLC: International Association for the Study of Lung Cancer
TTF-1: Thyroid transcription factor-1
MUC-2: Mucin-2
CD5: Cluster of differentiation 5
