# Supplementary material for: Non-mucinous, enteric-type thymic adenocarcinoma: genetic analysis of a case
Source: Gen Thorac Cardiovasc Surg Cases. 2026 Feb 2;5:8. doi: 10.1186/s44215-026-00240-x (PMC12952041; doi:10.1186/s44215-026-00240-x)
Supplement: Supplementary file 1 — Supplementary Material 1. [file 44215_2026_240_MOESM1_ESM.docx]

Supplementary Figure 1. Histopathological evidence of tumor invasion into the brachiocephalic vein, lung, and pericardium.

The tumor shows invasion into the adventitia of the brachiocephalic vein (a, hematoxylin and eosin [HE] , ×20; b, Elastica Van Gieson , ×20).

The tumor also shows invasion into the lung (c, H&E, ×40) and pericardium (d, H&E, ×40).
