# Supplementary material for: Non-mucinous, enteric-type thymic adenocarcinoma: genetic analysis of a case
Source: Gen Thorac Cardiovasc Surg Cases. 2026 Feb 2;5:8. doi: 10.1186/s44215-026-00240-x (PMC12952041; doi:10.1186/s44215-026-00240-x)
Supplement: Supplementary file 2 — Supplementary Material 2. [file 44215_2026_240_MOESM2_ESM.docx]

**Supplementary Table 2.** Clinicopathological characteristics of previously reported cases of enteric-type thymic adenocarcinoma.

| No. | Reference | Age | Sex | Size (cm) | CK20 | CDX-2 | CD5 | CK7 | MUC-2 |
| --- | --- | --- | --- | --- | --- | --- | --- | --- | --- |
| 1 | Wang et al[1]. | 70 | M | 7 × 4 × 3 | + | + | − | + | NA |
|  | *Int. J. Clin. Exp. Pathol. 2015* |  |  |  |  |  |  |  |  |
| 2 | Jung et al[2]. | 59 | F | 6.8 × 4.7 ×. 3.9 | + | + | + | +  (focal) | NA |
|  | *J. Pathol. Transl. Med. 2015* |  |  |  |  |  |  |  |  |
| 3 | Moser et al[3]. | 41 | M | 7.0 × 6.7 × 5.8 | + | + | − | − | NA |
|  | *Am. J. Surg. Pathol. 2015* |  |  |  |  |  |  |  |  |
| 4 | Moser et al[3]. | 39 | F | 4.3 × 4.0 × 3.9 | + | + | +  (focal) | + | NA |
| 5 | Maghbool et al[4] | 28 | F | NA | + | + | + | + | NA |
|  | *BMC Clin. Pathol. 2013* |  |  |  |  |  |  |  |  |
| 6 | Teramoto et al[5]. | 55 | M | 4.0 × 3.2 × 2.0 | + | + | + | + | NA |
|  | *Surg. Today 2012* |  |  |  |  |  |  |  |  |
| 7 | Abdul-Ghafar et al[6]. | 36 | F | 9 × 6 × 5 | + | +  (focal) | +  (focal) | + | NA |
|  | *Korean J. Pathol. 2012* |  |  |  |  |  |  |  |  |
| 8 | Seon et al[7]. | 66 | F | 11.0 × 9.5 × 9.0 | + | NA | + | NA | NA |
|  | *Int. J. Cardiol. 2012* |  |  |  |  |  |  |  |  |
| 9 | Weissferdt et al[8]. | 68 | M | 3.5 | NA | + | − | + | NA |
|  | *Am. J. Clin. Pathol. 2012* |  |  |  |  |  |  |  |  |
| 10 | Maeda et al[9]. | 52 | F | 9.5 × 6.0 × 5.5 | + | + | + | − | + |
|  | *Lung Cancer 2009* |  |  |  |  |  |  |  |  |
| 11 | Maeda et al[9]. | 38 | M | 8.0 × 7.0 × 3.5 | + | + | + | + | - |
| 12 | Maeda et al[9]. | 55 | M | 13.0 × 7.0 × 4.5 | + | + | + | − | + |
| 13 | Kapur et al[10]. | 41 | M | 10.5 × 8.0 × 2.5 | + | + | +  (focal) | +  (focal) | + |
|  | *Arch. Pathol. Lab. Med.* 2006 |  |  |  |  |  |  |  |  |
| 14 | Sawai et al[11]. | 34 | M | 5 × 4 | + | NA | + | − | NA |
|  | *Int. J. Surg. Pathol.2006* |  |  |  |  |  |  |  |  |
| 15 | Choi et al. | 15 | M | 8 × 5 × 4 | +  (focal) | NA | +  (focal) | +  (focal) | NA |
|  | *Am. J. Surg. Pathol. 2003* |  |  |  |  |  |  |  |  |
| 16 | Tamai et al[12]. | 29 | F | 12 × 11 | + | + | - | +  (focal) | - |
|  | *Diagn. Cytopathol. 2018* |  |  |  |  |  |  |  |  |
| 17 | Kinoshita et al[13]. | 79 | F | 9.4 × 7.6 × 5.1 | + | + | NA | - | NA |
|  | *Gen. Thorac. Cardiovasc. Surg. 2018* |  |  |  |  |  |  |  |  |
| 18 | Sakanoue et al[14]. | 39 | F | 6.5 × 3.5 | + | + | +  (focal) | + | NA |
|  | *J. Thorac. Dis. 2017* |  |  |  |  |  |  |  |  |
| 19 | Lee et al[15]. | 31 | M | 11.9 × 5.7 | + | + | +  (focal) | + | NA |
|  | *BMC Cancer 2017* |  |  |  |  |  |  |  |  |
| 20 | Haruki et al[16]. | 61 | F | 6.0 × 3.7 | + | + | NA | NA | NA |
|  | *J. Surg. Case Reports 2017* |  |  |  |  |  |  |  |  |
| 21 | Kwon et al[17]. | 70 | M | 9.6 | - | - | - | + | + |
|  | *Pathol. Res. Pract. 2017* |  |  |  |  |  |  |  |  |
| 22 | Kwon et al[17]. | 50 | M | 8.1 | + | + | - | - | + |
| 23 | Kwon et al[17]. | 62 | M | 3.2 | +  (focal) | + | - | - | + |
| 24 | Kwon et al[17]. | 56 | F | 7.6 | + | +  (focal) | +  (focal) | - | + |
| 25 | Kwon et al[17]. | 46 | M | 10 | + | + | +  (focal) | - | + |
| 26 | Kwon et al[17]. | 51 | M | 6.4 | NA | + | - | NA | NA |
| 27 | Kwon et al[17]. | 34 | M | 11.9 | + | + | - | + | NA |
| 28 | Kwon et al[17]. | 56 | F | 4.6 | + | - | - | - | + |
| 29 | Kwon et al[17]. | 57 | F | 5 | + | + | - | - | + |
| 30 | Fabio et al[18]. | 44 | M | NA | + | + | +  (focal) | - | NA |
|  | *Cancer Treat Rev*. 2021 |  |  |  |  |  |  |  |  |
| 31 | Fabio et al[18]. | 60 | M | 8 × 6 | +  (focal) | +  (focal) | NA | - | NA |
| 32 | Fabio et al[18]. | 24 | M | 3.0 × 3.3 × 3.5 | + | + | NA | +  (focal) | NA |
| 33 | Fabio et al[18]. | 45 | M | NA | + | + | - | - | NA |
| 34 | Hamanaka et al[19]. | 53 | F | 10.0 × 6.0 | + | + | - | - | NA |
|  | *Gen. Thorac. Cardiovasc.  Surg.* 2022 |  |  |  |  |  |  |  |  |
| 35 | Himuro et al[20]. | 58 | F | 10.0 × 6.0 | + | + | - | +  (focal) | NA |
|  | *Clin. Lung Cancer*. 2021 |  |  |  |  |  |  |  |  |

NA: not available

**References**

1. Wang L, Wang D, Qian K, Lu D, Chen L, Zhao L, et al. Thymic adenocarcinoma associated with thymic cyst: a case report and review of literature. Int J Clin Exp Pathol 2015; 8:5890–5.
2. Jung HY, Cho H, Chung JH, Bae SB, Lee JH, Lee HJ, et al. A rare case of primary tubular adenocarcinoma of the thymus, enteric immunophenotype: a case study and review of the literature. J Pathol Transl Med 2015; 49:331–4.
3. Moser B, Schiefer AI, Janik S, Marx A, Prosch H, Pohl W, et al. Adenocarcinoma of the thymus, enteric type: report of 2 cases, and proposal for a novel subtype of thymic carcinoma. Am J Surg Pathol 2015; 39:541–8.
4. Maghbool M, Ramzi M, Nagel I, Bejarano P, Siebert R, Saeedzadeh A, et al. Primary adenocarcinoma of the thymus: an immunohistochemical and molecular study with review of the literature. BMC Clin Pathol. 2013 May 31;13(1):17.
5. Teramoto K, Kawaguchi Y, Hori T, Hashimoto M, Kitamura S, Motoishi M, et al. Thymic papillo-tubular adenocarcinoma containing a cyst: report of a case. Surg Today 2012; 42:988–91.
6. Abdul-Ghafar J, Yong SJ, Kwon W, Park IH, Jung SH. Primary thymic mucinous adenocarcinoma: a case report. Korean J Pathol. 2012 Aug;46(4):377-81.
7. Seon HJ, Kim KH, Choi YD, Song SY, Yoon HJ, Kim YH, Jeong MH, Park JC. Angina pectoris caused by the extrinsic compression of coronary artery by primary thymic mucinous adenocarcinoma. Int J Cardiol. 2012 Apr 5;156(1):e13-5.
8. Weissferdt A, Moran CA. Thymic carcinoma, part 1: a clinicopathologic and immunohistochemical study of 65 cases. Am J Clin Pathol. 2012 Jul;138(1):103-14.
9. Maeda D, Ota S, Ikeda S, Kawano R, Hata E, Nakajima J, Mori M, Fukayama M. Mucinous adenocarcinoma of the thymus: a distinct variant of thymic carcinoma. Lung Cancer. 2009 Apr;64(1):22-7.
10. Kapur P, Rakheja D, Bastasch M, Molberg KH, Sarode VR. Primary mucinous adenocarcinoma of the thymus: a case report and review of the literature. Arch Pathol Lab Med. 2006 Feb;130(2):201-4.
11. Sawai T, Inoue Y, Doi S, Ikuta Y, Kimino K, Nakashima M, et al. Tubular adenocarcinoma of the thymus: case report and review of the literature. Int J Surg Pathol 2006; 14:243–6.
12. Tamai M, Ishida M, Ebisu Y, Okamoto Y, Miyasaka C, Ohe C, et al. Thymic enteric type adenocarcinoma: a case report with cytological features. Diagn Cytopathol 2018; 46:92–7.
13. Kinoshita F, Shoji F, Takada K, Toyokawa G, Okamoto T, Yano T, et al. Mucinous adenocarcinoma of the thymus: report of a case. Gen Thorac Cardiovasc Surg. 2018 Feb;66(2):111-115.
14. Sakanoue I, Hamakawa H, Fujimoto D, Imai Y, Minami K, Tomii K, et al. KRAS mutation-positive mucinous adenocarcinoma originating in the thymus. J Thorac Dis 2017; 9:E694–7.
15. Lee Y, Park S, Lee SH, Lee H. Characterization of genetic aberrations in a single case of metastatic thymic adenocarcinoma. BMC Cancer 2017; 17:330.
16. Haruki T, Wakahara M, Taniguchi Y, Nakamura Y, Nishimura M, Nakamura H. Successful multimodality treatment for locally advanced primary thymic adenocarcinoma: report of a case. J Surg Case Rep. 2016 Aug 31;2016(8):rjw148.
17. Kwon AY, Han J, Chu J, Choi Y, Jeong B, Ahn M, et al. Histologic characteristics of thymic adenocarcinomas: clinicopathologic study of a nine-case series and a review of the literature. Pathol Res Pract 2017; 213:106–12.
18. Conforti F, Tarantino P, Trillo P, Pala L, Zagami P, Pirola S, et al. Pathological and clinical features of enteric adenocarcinoma of the thymus. A pooled analysis of cases from a reference center and systematic review of the literature. Cancer Treat Rev 2021; 92:102133.
19. Hamanaka R, Nakano K, Tsuboi T, Hatanaka K, Kohno M, Masuda R, et al. Enteric-type thymic adenocarcinoma: a case report and literature review focusing on prognosis based on histological subtypes. Gen Thorac Cardiovasc Surg 2022; 70:501–5.
20. Himuro N, Kitami A, Tanaka Y, Ohashi S, Suzuki K, Ohara J, et al. STK11/LKB1 mutation-positive primary thymic mucinous adenocarcinoma accompanied by cerebellar metastasis: a case report and literature review. Clin Lung Cancer 2021; 22:e659–64.
